# Crystal structure, Hirshfeld surface analysis, crystal voids, inter­action energy calculations and energy frameworks, and DFT calculations of 1-(4-methyl­benz­yl)in­do­line-2,3-dione

**DOI:** 10.1107/S2056989024000756

**Published:** 2024-01-31

**Authors:** Nohaila Rharmili, Omar Abdellaoui, Fouad Ouazzani Chahdi, Joel T. Mague, Tuncer Hökelek, Ahmed Mazzah, Youssef Kandri Rodi, Nada Kheira Sebbar

**Affiliations:** a Laboratory of Applied Organic Chemistry, Sidi Mohamed Ben Abdellah University, Faculty Of Science And Technology, Road Immouzer, BP 2202 Fez, Morocco; b Department of Chemistry, Tulane University, New Orleans, LA 70118, USA; c Department of Physics, Hacettepe University, 06800 Beytepe, Ankara, Türkiye; d Science and Technology of Lille USR 3290, Villeneuve d’ascq cedex, France; e Laboratory of Organic and Physical Chemistry, Applied Bioorganic Chemistry Team, Faculty of Sciences, Ibnou Zohr University, Agadir, Morocco; f Laboratory of Plant Chemistry, Organic and Bioorganic Synthesis, Faculty of Sciences, Mohammed V University in Rabat, 4 Avenue Ibn Battouta B.P. 1014 RP, Rabat, Morocco; Tokyo University of Science, Japan

**Keywords:** hydrogen bonds, C—H⋯π(ring) inter­action, π-stacking, C=O⋯π(ring) inter­action, in­do­line-2,3-dione, crystal structure

## Abstract

The in­do­line portion of the title mol­ecule, C_16_H_13_NO_2_, is planar. In the crystal, a layer structure is generated by C—H⋯O hydrogen bonds and C—H⋯π(ring), π-stacking and C=O⋯π(ring) inter­actions. The Hirshfeld surface analysis of the crystal structure indicates that the most important contributions for the crystal packing are from H⋯H (43.0%), H⋯C/C⋯H (25.0%) and H⋯O/O⋯H (22.8%) inter­actions. Hydrogen bonding and van der Waals inter­actions are the dominant inter­actions in the crystal packing. The volume of the crystal voids and the percentage of free space were calculated to be 120.52 Å^3^ and 9.64%, respectively, showing that there is no large cavity in the crystal packing. Evaluation of the electrostatic, dispersion and total energy frameworks indicate that the stabilization is dominated by the dispersion energy contributions in the title compound. Moreover, the DFT-optimized structure at the B3LYP/6-311G(d,p) level is compared with the experimentally determined mol­ecular structure in the solid state.

## Abstract 2

In the crystal of 1-(4-methyl­benz­yl)in­do­line-2,3-dione, a layer structure is generated by C—H⋯O hydrogen bonds and C—H⋯π(ring), π-stacking and C=O⋯π(ring) inter­actions.

## 1. Chemical context

Isatin derivatives have a biologically active heterocyclic moiety that comprises two cyclic rings, one of which is six-membered and the other is five-membered (Rharmili *et al.*, 2023*a* ▸). Both the rings are planar. It constitutes an important class of heterocyclic compounds which, even when part of a complex mol­ecule, possess a wide spectrum of biological activities (Rharmili *et al.*, 2023*b* ▸), such as anti­cancer (Esmaeelian *et al.*, 2013 ▸), anti­oxidant (Andreani *et al.*, 2010 ▸), anti­malarial (Chiyanzu *et al.*, 2005 ▸), anti-inflammatory (Sharma *et al.*, 2016 ▸), analgesic (Prakash *et al.*, 2012 ▸) and anti-anxiety (Medvedev *et al.*, 2005 ▸). They have also been studied and been reported as efficient inhibitors against aluminium and steel corrosion (Abdellaoui *et al.*, 2021 ▸). In a continuation of our ongoing research work devoted to the study of *O*-alkyl­ation and *N*-alkyl­ation reactions involving isatin derivatives (Rharmili *et al.*, 2023*b* ▸), we report herein the synthesis and the mol­ecular and crystal structures of 1-(4-methyl­benz­yl)in­do­line-2,3-dione (Scheme 1) obtained by an alkyl­ation reaction of 1*H*-in­do­line-2,3-dione using an excess of 4-methyl­benzyl bromide as an alkyl­ating reagent and potassium carbonate in the presence of tetra-*n*-butyl­ammonium bromide as catalyst in phase-transfer catalysis (PTC). Moreover, a Hirshfeld surface analysis, crystal voids, and inter­action energy and energy frameworks calculations were performed. The mol­ecular structure optimized by density functional theory (DFT) at the B3LYP/6-311G(d,p) level is compared with the experimentally determined mol­ecular structure in the solid state. 
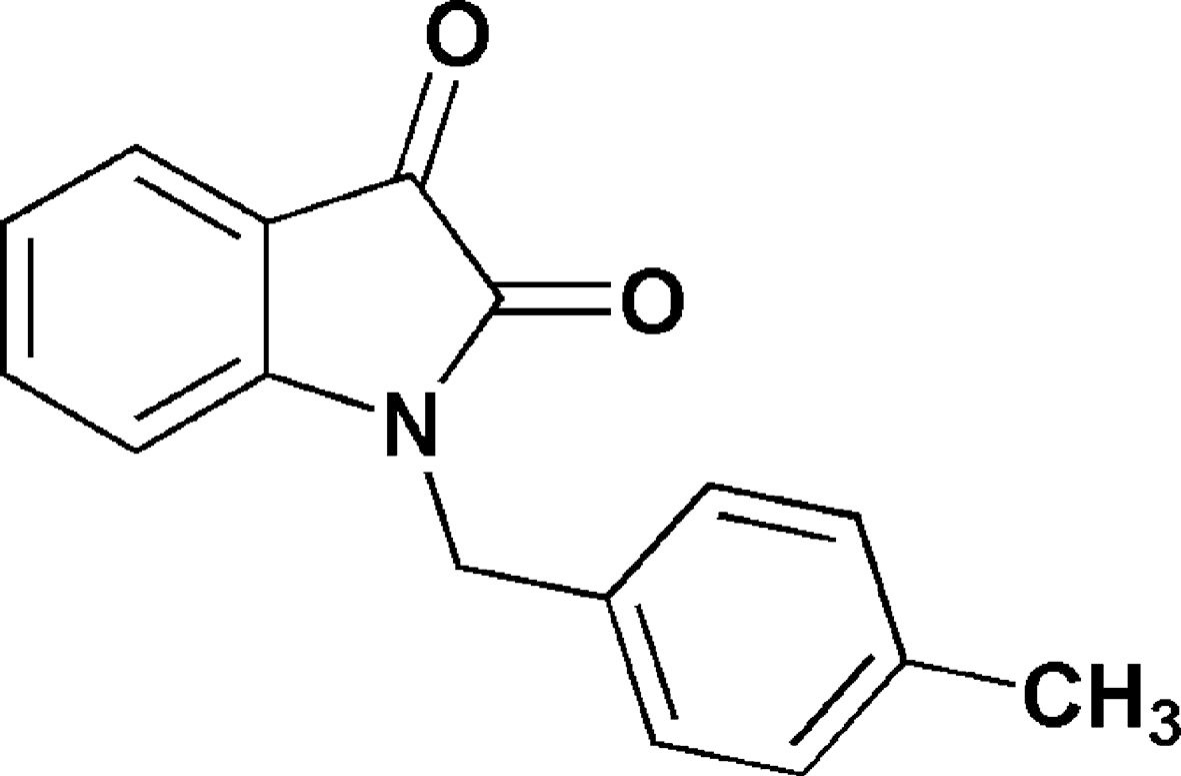


## 2. Structural commentary

**Figure 1:**
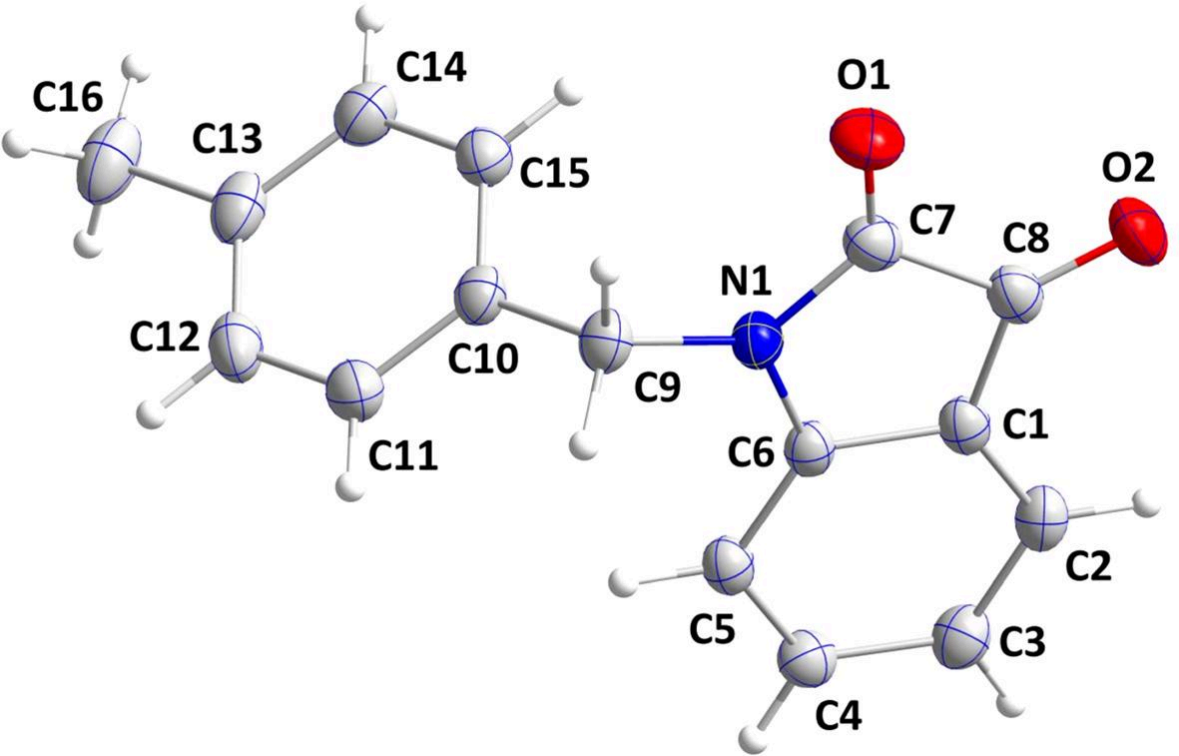
The title mol­ecule with the atom-labelling scheme and 50% probability displacement ellipsoids.

The in­do­line portion (Fig. 1 ▸) is planar to within 0.0097 (10) Å (r.m.s. deviation of the fitted atoms = 0.0050 Å) and the mean plane of the C10–C15 ring is inclined to the above plane by 79.03 (3)°. The C7—C8 bond, at 1.5555 (18) Å, is longer than expected for that between two *sp* ^2^ C atoms but apppears typical for in­do­line-2,3-diones. Otherwise, the metrical parameters are unremarkable.

## 3. Supra­molecular features

**Figure 2:**
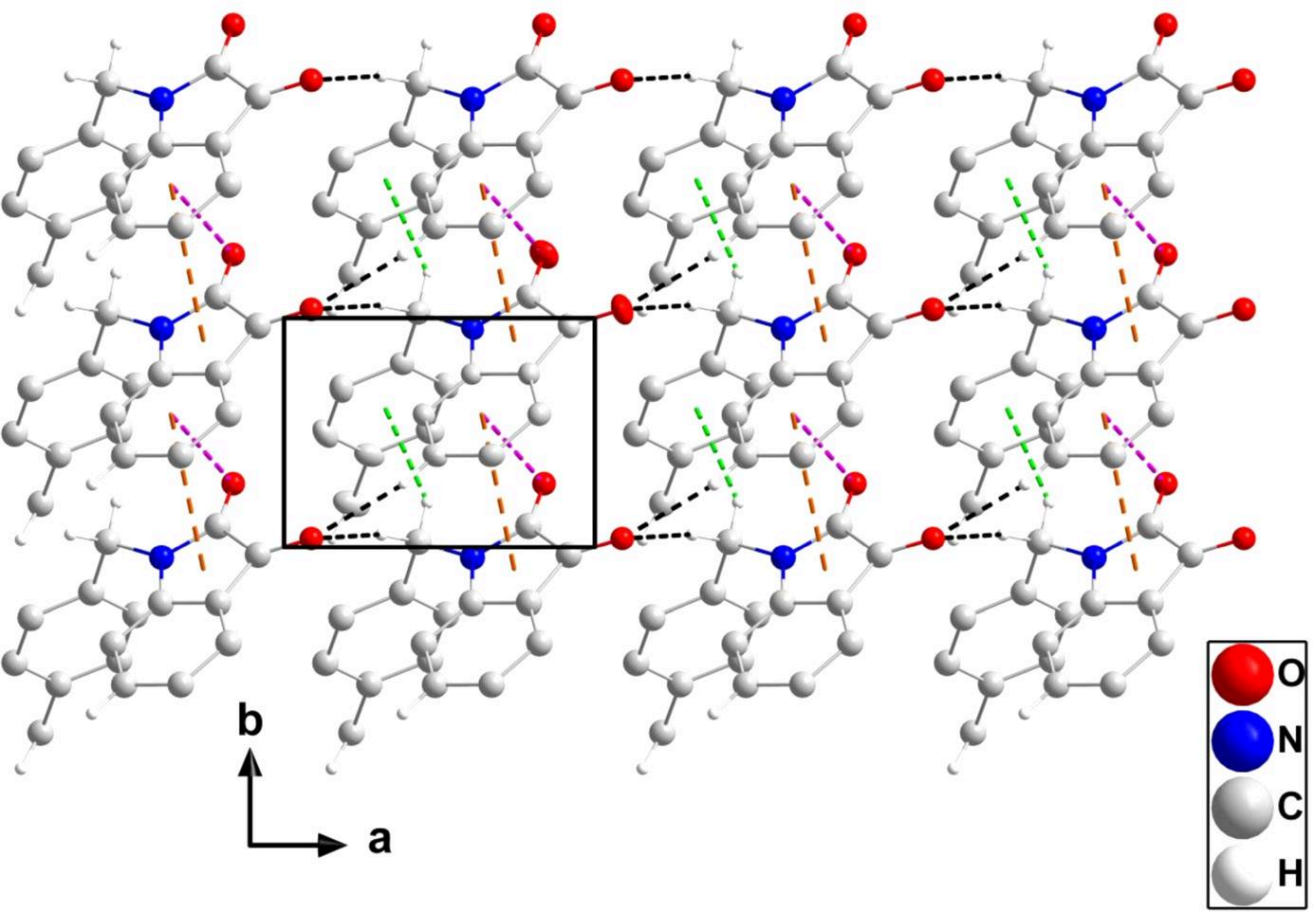
A portion of one layer, viewed along the *c*-axis direction, with C—H⋯O hydrogen bonds and C—H⋯π(ring) and π-stacking inter­actions depicted, respectively, by black, green and orange dashed lines. The C= O⋯π(ring) inter­actions are depicted by pink dashed lines and non-inter­acting H atoms have been omitted for clarity.

**Figure 3:**
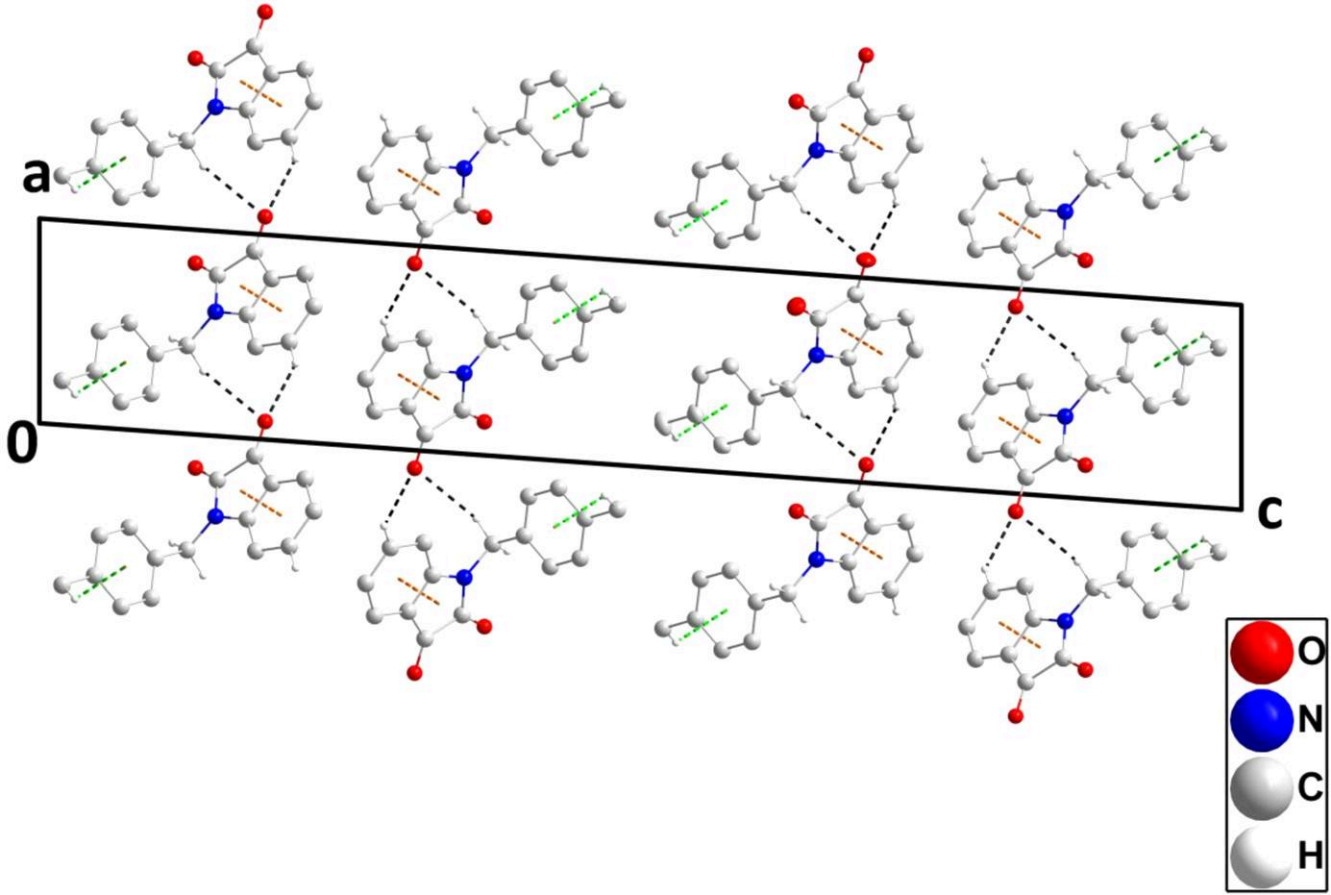
The packing viewed along the *b*-axis direction giving edge views of four layers. C—H⋯O hydrogen bonds and C—H⋯π(ring) and π-stacking inter­actions are depicted, respectively, by black, green and orange dashed lines, while the C=O⋯π(ring) inter­actions and non-inter­acting H atoms have been omitted for clarity.

In the crystal, C9—H9*B*⋯O2^iii^ hydrogen bonds (Table 1 ▸) form chains of mol­ecules extending along the *a*-axis direction which are elaborated along the *b*-axis direction by C4—H4⋯O2^i^ hydrogen bonds (Table 1 ▸) to form layers parallel to the *ab* plane (Fig. 2 ▸). The layer formation is reinforced by C9—H9*A*⋯*Cg*3^ii^ and C16—H16*B*⋯*Cg*3^iv^ inter­actions (Table 1 ▸), as well as slipped π-stacking inter­actions between the C1–C6 and C1/C6/N1/C7/C8 rings related by unit translations along the *b*-axis direction [centroid–centroid = 3.6004 (8) Å, dihedral angle = 0.42 (6)° and slippage = 1.39 Å, where *Cg*3 is the centroid of the C10–C15 benzene ring]. Also present are C7=O1⋯*Cg*1 inter­actions in the same direction [*Cg*1 is the centroid of the C1–C6 ring; O1⋯*Cg*1 = 3.4793 (12) Å, C7⋯*Cg*1 = 4.0442 (15) Å and C7=O1⋯*Cg*1 = 109.34 (9)°]. A portion of one layer is shown in Fig. 2 ▸, while the packing of the layers is shown in Fig. 3 ▸.

## 4. Hirshfeld surface analysis

**Figure 4:**
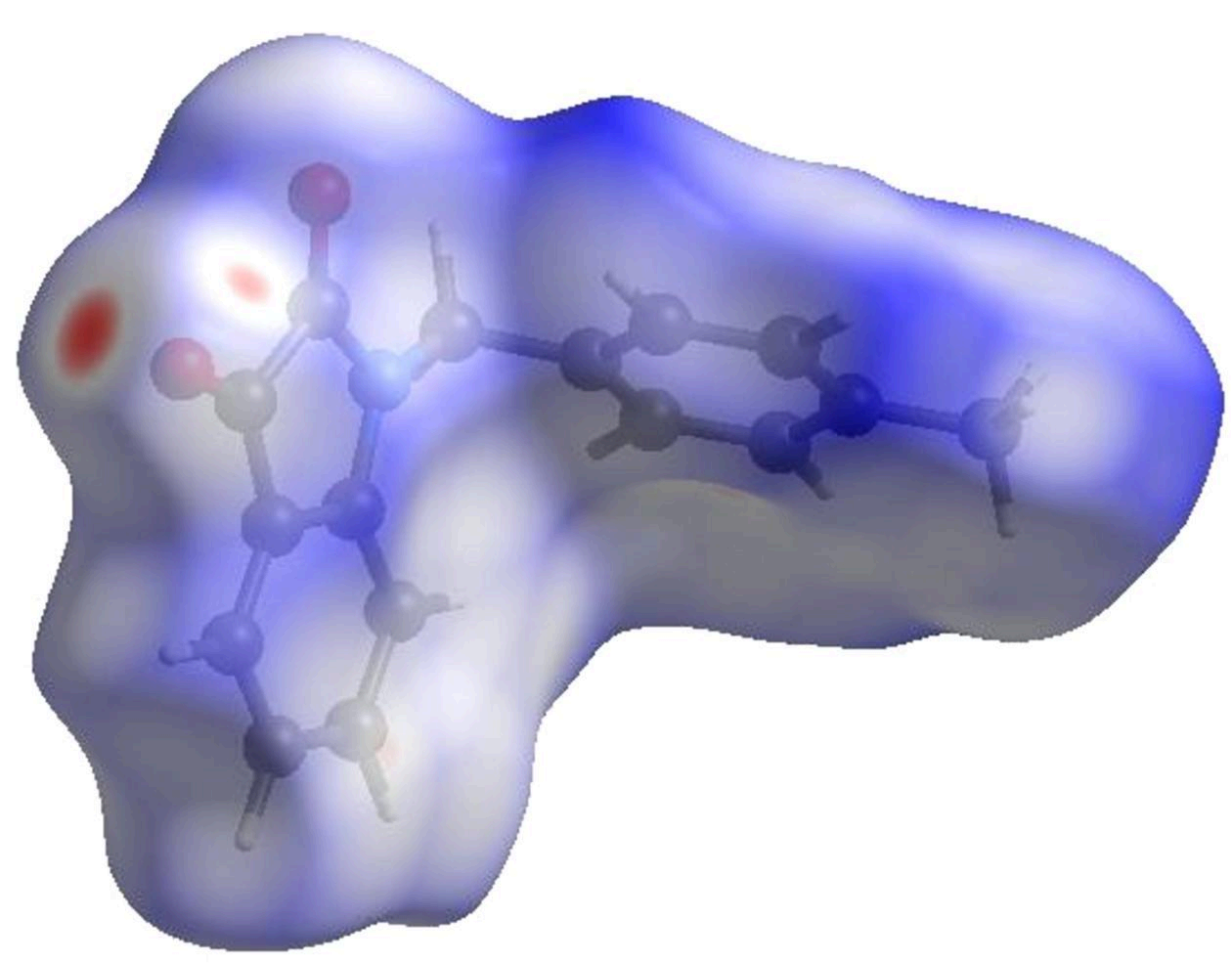
View of the three-dimensional Hirshfeld surface of the title compound plotted over *d*_norm_.

**Figure 5:**
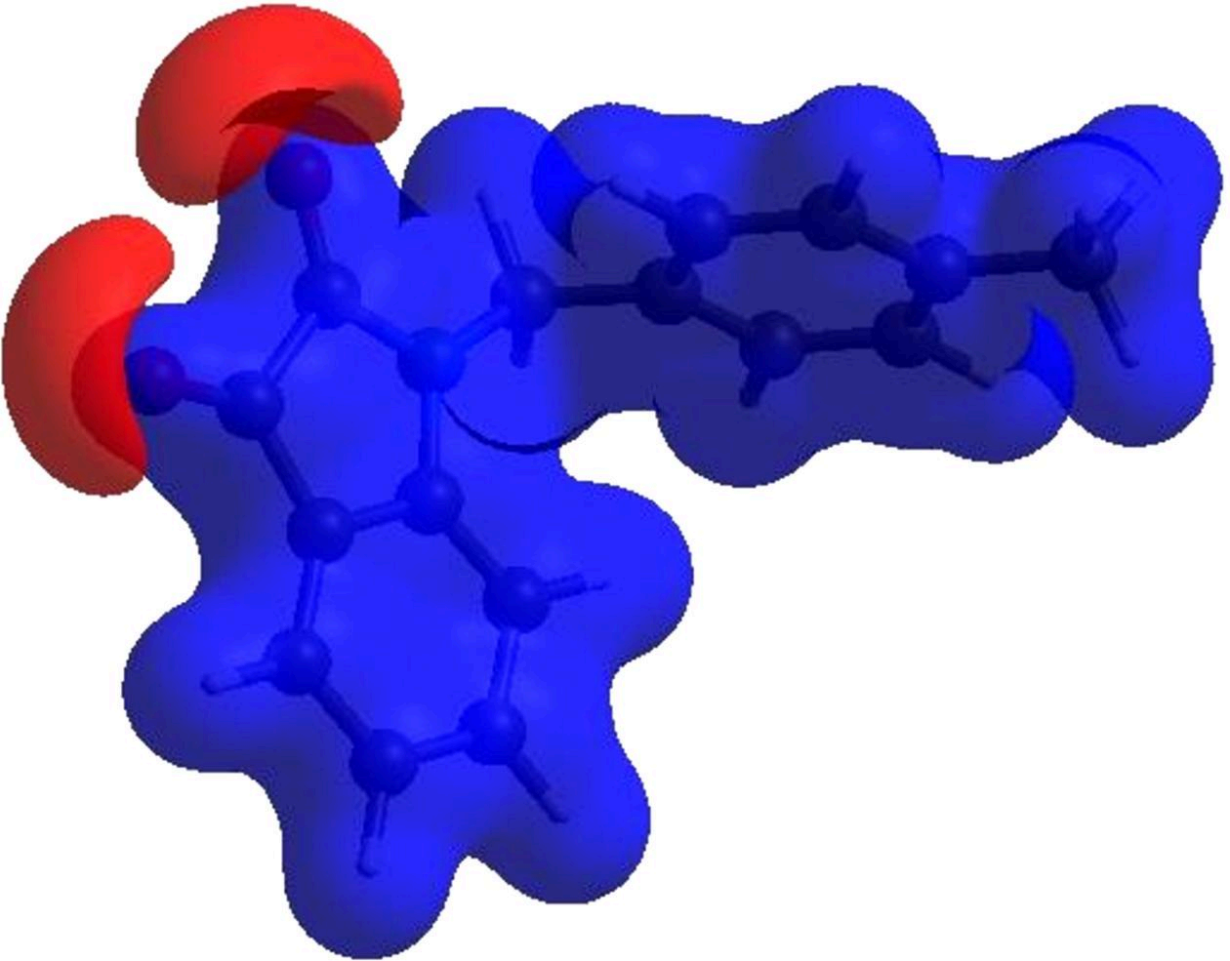
View of the three-dimensional Hirshfeld surface of the title compound plotted over electrostatic potential energy using the STO-3 G basis set at the Hartree–Fock level of theory. Hydrogen-bond donors and acceptors are shown as blue and red regions around the atoms corresponding to positive and negative potentials, respectively.

**Figure 6:**
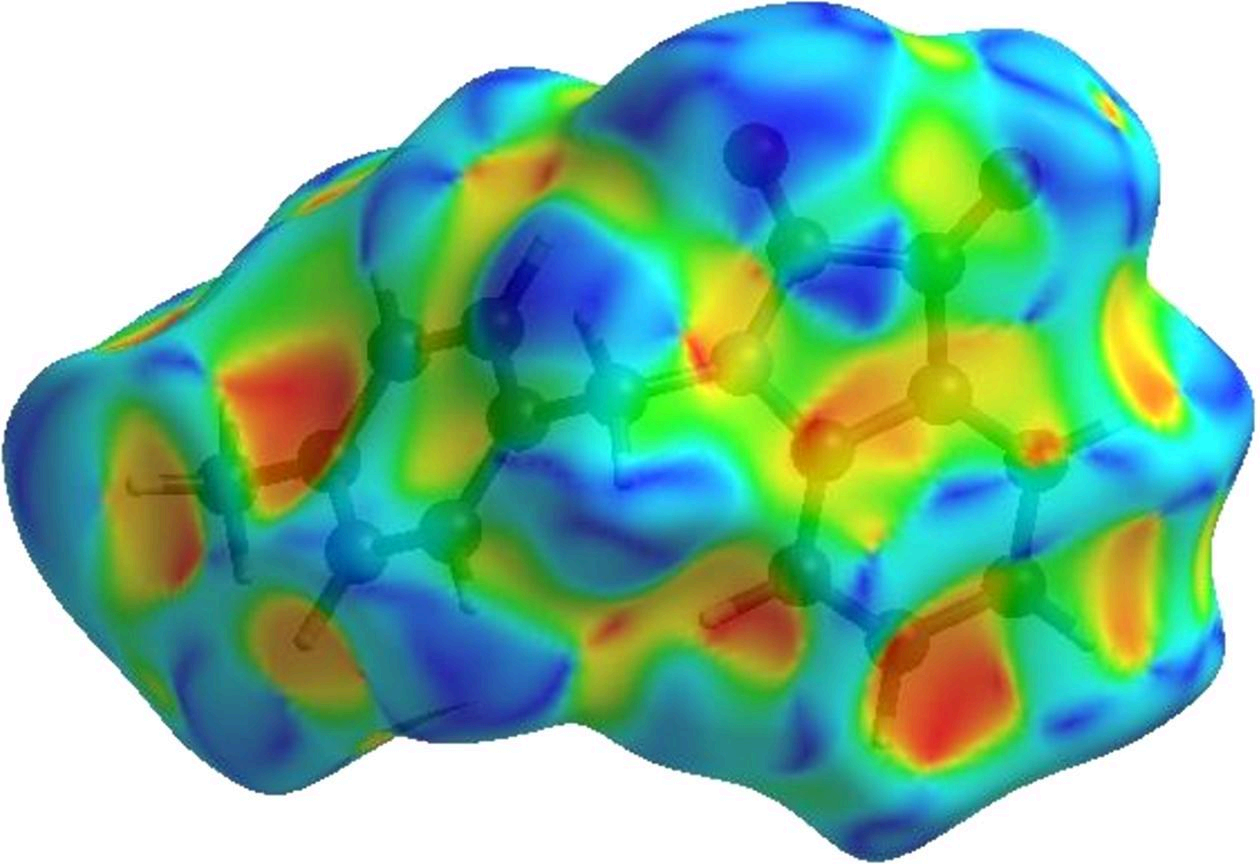
Hirshfeld surface of the title compound plotted over shape index.

**Figure 7:**
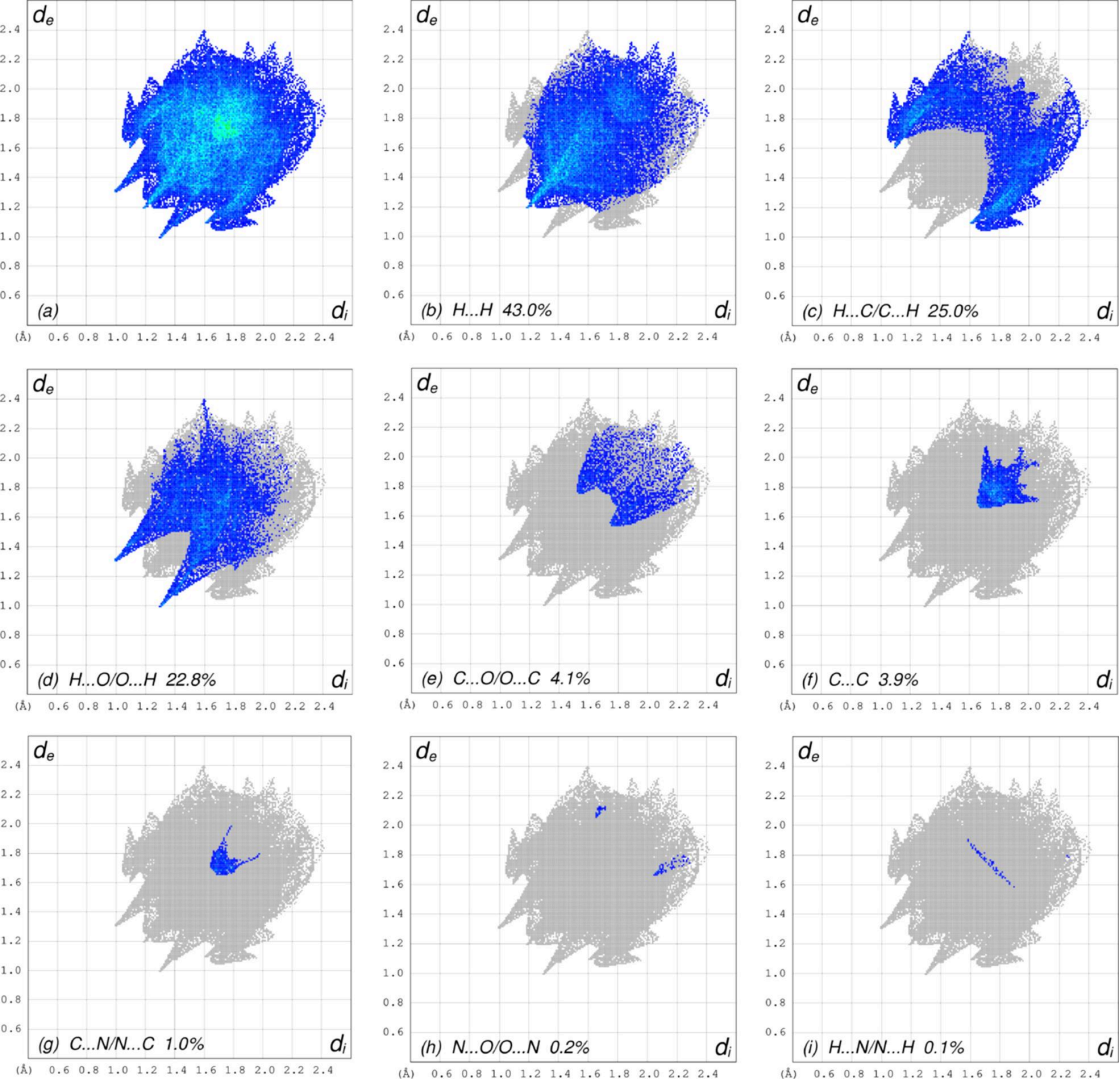
The full two-dimensional fingerprint plots for the title compound, showing (*a*) all inter­actions, and delineated into (*b*) H⋯H, (*c*) H⋯C/C⋯H, (*d*) H⋯O/O⋯H, (*e*) C⋯O/O⋯C, (*f*) C⋯C, (*g*) C⋯N/N⋯C, (*h*) N⋯O/O⋯N and (*i*) H⋯N/N⋯H inter­actions. The *d*_i_ and *d*_e_ values are the closest inter­nal and external distances (in Å) from given points on the Hirshfeld surface contacts.

In order to visualize the inter­molecular inter­actions in the crystal of the title compound, (I), a Hirshfeld surface (HS) analysis (Hirshfeld, 1977 ▸; Spackman & Jayatilaka, 2009 ▸) was carried out using *CrystalExplorer* (Version 17.5; Turner *et al.*, 2017 ▸). In the HS plotted over *d*_norm_ (Fig. 4 ▸), the white surface indicates contacts with distances equal to the sum of the van der Waals radii, and the red and blue colours indicate distances shorter (in close contact) or longer (distinct contact) than the van der Waals radii, respectively (Venkatesan *et al.*, 2016 ▸). The bright-red spots indicate their roles as the respective donors and/or acceptors; they also appear as blue and red regions corresponding to positive and negative potentials on the HS mapped over electrostatic potential (Spackman *et al.*, 2008 ▸; Jayatilaka *et al.*, 2005 ▸), as shown in Fig. 5 ▸. The blue regions indicate the positive electrostatic potential (hydrogen-bond donors), while the red regions indicate the negative electrostatic potential (hydrogen-bond acceptors). The shape index of the HS is a tool to visualize the π–π stacking by the presence of adjacent red and blue triangles; if there are no adjacent red and/or blue triangles, then there are no π–π inter­actions. Fig. 6 ▸ clearly suggests that there are π–π inter­actions in (I). The overall two-dimensional fingerprint plot, Fig. 7 ▸(*a*), and those delineated into H⋯H, H⋯C/C⋯H, H⋯O/O⋯H, C⋯O/O⋯C, C⋯C, N⋯C/C⋯N, N⋯O/O⋯N and H⋯N/N⋯H (McKinnon *et al.*, 2007 ▸) are illustrated in Figs. 7 ▸(*b*)–(*i*), respectively, together with their relative contributions to the Hirshfeld surface. The most abundant inter­action is H⋯H, contributing 43.0% to the overall crystal packing, which is reflected in Fig. 7 ▸(*b*) as the widely scattered points of high density due to the large hydrogen content of the mol­ecule with the tip at *d*_e_ = *d*_i_ = 1.20 Å. In the presence of C—H⋯π inter­actions, the H⋯C/C⋯H contacts, contributing 25.0% to the overall crystal packing, are reflected in Fig. 7 ▸(*c*) with the tips at *d*_e_ + *d*_i_ = 2.71 Å. The symmetrical pair of spikes resulting in the fingerprint plot delineated into H⋯O/O⋯H contacts [Fig. 7 ▸(*d*)] has a 22.8% contribution to the HS with the tips at *d*_e_ + *d*_i_ = 2.29 Å. The symmetrical pair of tiny wings resulting in the fingerprint plot delineated into C⋯O/O⋯C contacts [Fig. 7 ▸(*e*)], with a 4.1% contribution to the HS, is viewed with the tips at *d*_e_ + *d*_i_ = 3.29 Å. The C⋯C contacts [Fig. 7 ▸(*f*)] have an arrow-shaped distribution of points, with the tip at *d*_e_ = *d*_i_ = 1.68 Å. Finally, the C⋯N/N⋯C [Fig. 7 ▸(*g*)], N⋯O/O⋯N [Fig. 7 ▸(*h*)] and H⋯N/N⋯H [Fig. 7 ▸(*i*)] contacts with 1.0, 0.2 and 0.1% contributions, respectively, to the HS have very low distributions of points.

**Figure 8:**
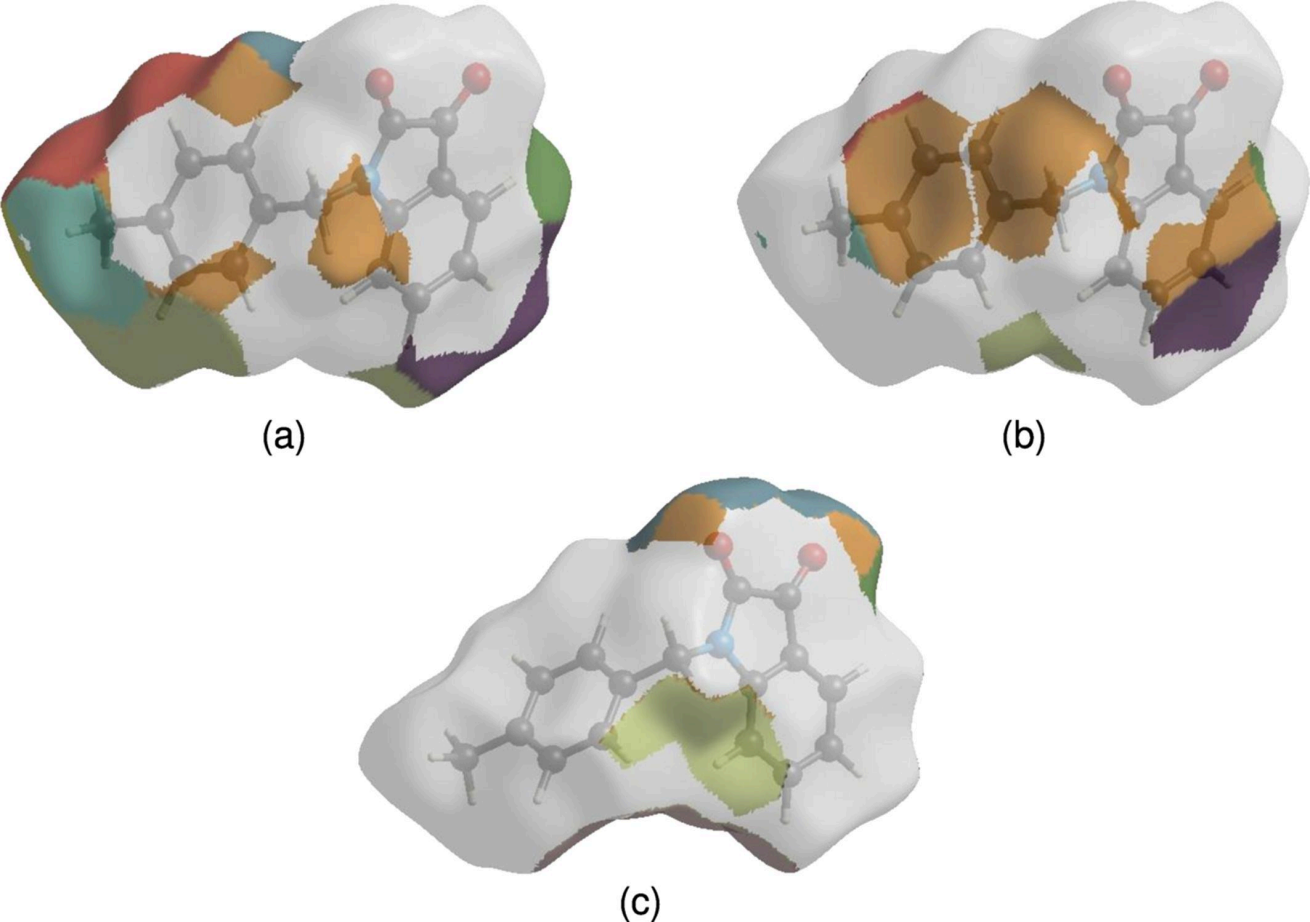
The Hirshfeld surface representations with the function fragment patch plotted onto the surface for (*a*) H⋯H, (*b*) H⋯C/C⋯H and (*c*) H⋯O/O⋯H inter­actions.

The nearest-neighbour coordination environment of a mol­ecule can be determined from the colour patches on the HS based on how close to other mol­ecules they are. The Hirshfeld surface representations with the function *d*_norm_ plotted onto the surface are shown for the H⋯H, H⋯C/C⋯H and H⋯N/N⋯H inter­actions in Figs. 8 ▸(*a*)–(*c*), res­pectively. The Hirshfeld surface analysis confirms the importance of H-atom contacts in establishing the packing. The large number of H⋯H, H⋯C/C⋯H and H⋯N/N⋯H inter­actions suggest that van der Waals inter­actions and hydrogen bonding play the major roles in the crystal packing (Hathwar *et al.*, 2015 ▸).

## 5. Crystal voids

**Figure 9:**
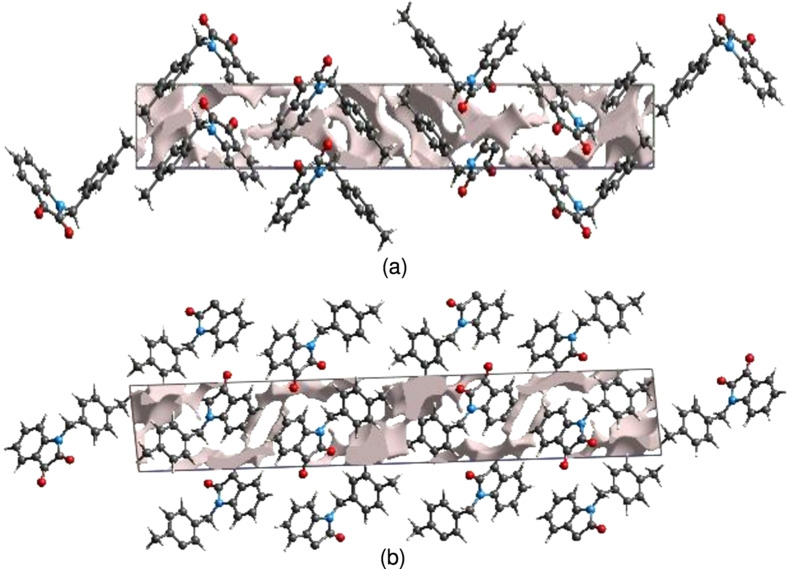
Graphical views of voids in the crystal packing of (I) (*a*) along the *a*-axis direction and (*b*) along the *b*-axis direction.

The strength of the crystal packing is important for determining the response to an applied mechanical force. If the crystal packing results in significant voids, then the mol­ecules are not tightly packed and a small amount of applied external mechanical force may easily break the crystal. For checking the mechanical stability of the crystal, a void analysis was performed by adding up the electron densities of the spherically symmetric atoms contained in the asymmetric unit (Turner *et al.*, 2011 ▸). The void surface is defined as an isosurface of the procrystal electron density and is calculated for the whole unit cell where the void surface meets the boundary of the unit cell and capping faces are generated to create an enclosed volume. The volume of the crystal voids [Figs. 9 ▸(*a*) and 9(*b*)] and the percentage of free space in the unit cell are calculated as 120.52 Å^3^ and 9.64%, respectively. Thus, the crystal packing appears compact and the mechanical stability should be substantial.

## 6. Inter­action energy calculations and energy frameworks

**Figure 10:**
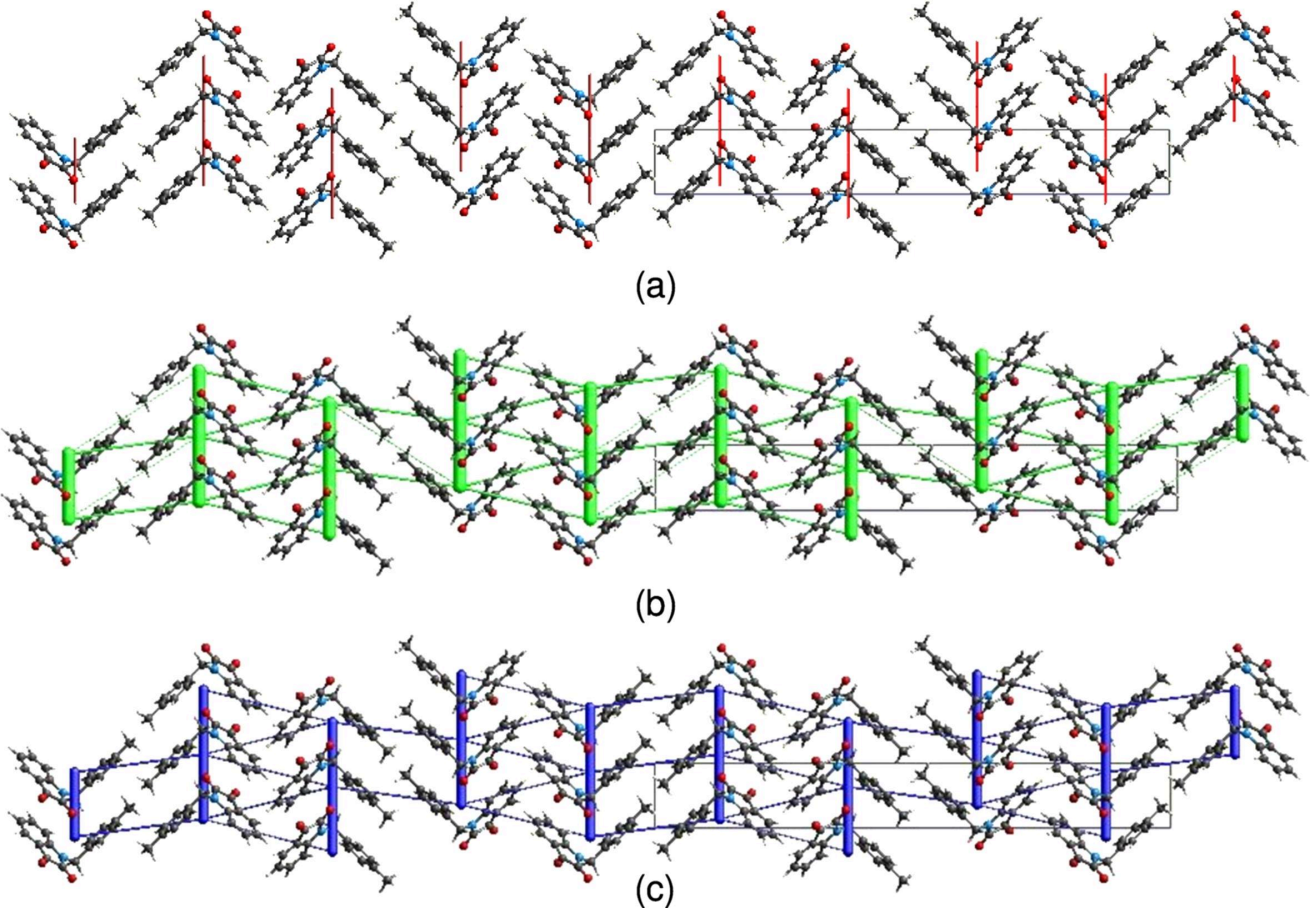
The energy frameworks for a cluster of mol­ecules of the title compound, viewed down the *a*-axis direction, showing (*a*) electrostatic energy, (*b*) dispersion energy and (*c*) total energy diagrams. The cylindrical radius is proportional to the relative strength of the corresponding energies and they were adjusted to the same scale factor of 80 with a cut-off value of 5 kJ mol^−1^ within 2 × 2 × 2 unit cells.

The inter­molecular inter­action energies are calculated using the CE-B3LYP/6-31G(d,p) energy model available in *CrystalExplorer* (Version 17.5; Turner *et al.*, 2017 ▸), where a cluster of mol­ecules is generated by applying crystallographic symmetry operations with respect to a selected central mol­ecule within the radius of 3.8 Å by default (Turner *et al.*, 2014 ▸). The total inter­molecular energy (*E*_tot_) is the sum of electrostatic (*E*_ele_), polarization (*E*_pol_), dispersion (*E*_dis_) and exchange–repulsion (*E*_rep_) energies (Turner *et al.*, 2015 ▸), with scale factors of 1.057, 0.740, 0.871 and 0.618, respectively (Mackenzie *et al.*, 2017 ▸). Hydrogen-bonding inter­action energies (in kJ mol^−1^) were calculated to be [−11.6 (*E*_ele_), −4.3 (*E*_pol_), −71.9 (*E*_dis_), 46.4 (*E*_rep_) and −49.4 (*E*_tot_)] for the C4—H4⋯O2 and [−5.4 (*E*_ele_), −3.9 (*E*_pol_), −24.7 (*E*_dis_), 14.3 (*E*_rep_) and −21.3 (*E*_tot_)] for the C9—H9*B*⋯O2 hydrogen-bonding inter­action. Energy frameworks combine the calculation of inter­molecular inter­action energies with a graphical representation of their magnitude (Turner *et al.*, 2015 ▸). Energies between mol­ecular pairs are represented as cylinders joining the centroids of pairs of mol­ecules with the cylinder radius proportional to the relative strength of the corresponding inter­action energy. Energy frameworks were constructed for *E*_ele_ (red cylinders), *E*_dis_ (green cylinders) and *E*_tot_ (blue cylinders) [Figs. 10 ▸(*a*), 10(*b*) and 10(*c*)]. The evaluation of the electrostatic, dispersion and total energy frameworks indicate that the stabilization is dominated *via* the dispersion energy contribution in the crystal structure of (I).

## 7. Database survey

We searched the Cambridge Structural Database (CSD) for *N*-substituted isatin derivatives using Version 5.42, which was last updated in May 2023 (Groom *et al.*, 2016 ▸). Our search yielded 58 results, five of which were reports on the structure of isatin itself, and four of which focused on the structure of *N*-methyl­isatin. Out of these findings, 13 structures contained an alkyl chain with two or more C atoms. The compound that showed the closest resemblance to the title compound was indole-2,3-dione (Wang *et al.*, 2010 ▸).

## 8. DFT calculations

The gas-phase mol­ecular structure was theoretically optimized using density functional theory (DFT) with the B3LYP functional and 6-311++G(d,p) basis-set calculations (Becke, 1993 ▸) as implemented in *GAUSSIAN09* (Frisch *et al.*, 2009 ▸). The resulting optimized parameters, including bond lengths and angles, exhibited satisfactory agreement with the experimental structural data (Table 2 ▸). The most significant disparities between the calculated and experimental values were observed for the O1—C7 and N1—C9 (0.04 Å), and C1—C2 and O2—C8 (0.03 Å) bond lengths. Additionally, notable disparities were noted in the O1—C7—C8 bond angle (3.05°) and the C7—N1—C9—C10 torsion angle (0.85°). For instance, some reported bond lengths for O1—C7 and N1—C9 were fuond to vary by 0.03 and 0.01 Å, respectively, for 1-(12-bromo­dodec­yl)in­do­line-2,3-dione (Rharmili *et al.*, 2023*a* ▸). These differences may be attributed to the fact that these calculations pertain to the isolated mol­ecule, while the experimental results correspond to inter­acting mol­ecules in the crystal lattice, where intra- and inter­molecular inter­actions with neighbouring mol­ecules are present.

## 9. Synthesis and crystallization

To a solution of 1*H*-in­do­line-2,3-dione (2 mmol) in di­methyl­formamide (DMF, 20 ml) were added 4-methyl­benzyl bromide (2.2 mmol), K_2_CO_3_ (1.5 mmol) and tetra-*n*-butyl­ammonium bromide (TBAB; 0.5 mmol). The reaction mixture was stirred at room temperature in DMF for 12 h. After removal of the formed salts, the solvent was evaporated under reduced pressure and the residue obtained was dissolved in di­chloro­methane. The organic phase was dried over Na_2_SO_4_ and then concentrated *in vacuo*. A pure compound was obtained after recrystallization from ethanol/hexane (3:1 *v*/*v*) (yield 92%; m.p. 356 K). ^1^H NMR (300 MHz, *d*_6_-DMSO): δ 7.62 (2H, *m*); 7.33 (2H, *m*); 7.18 (3H, *dt*, ^3^ *J* = 8.4 Hz); 6.97 (1H, *t*, ^3^ *J* = 7.5 Hz); 4.86 (2H, *s*); 2.27 (3H, *s*). ^13^C NMR (75 MHz, *d*_6_-DMSO): δ 183.62 (–C=O); 158.73 (N—C=O); 150.83 (C_q_); 140.47 (CH_Ar_); 138.44 (CH_Ar_); 137.42(C_q_); 133.48 (CH_Ar_); 132.90 (C_q_); 129.27 (CH_Ar_); 127.85 (CH_Ar_); 126.61 (CH_Ar_); 126.56 (CH_Ar_); 124.94 (C_q_); 123.78 (CH_Ar_); 43.7 (CH_2_); 21.13 (CH_3_).

## 10. Refinement

Crystal data, data collection and structure refinement details are summarized in Table 3 ▸. H atoms attached to carbon were placed in calculated positions (C—H = 0.95–0.99 Å). All were included as riding contributions with isotropic displacement parameters 1.2–1.5 times those of the attached atoms.

## Supplementary Material

Crystal structure: contains datablock(s) global, I. DOI: 10.1107/S2056989024000756/jp2002sup1.cif

Structure factors: contains datablock(s) I. DOI: 10.1107/S2056989024000756/jp2002Isup2.hkl

Supporting information file. DOI: 10.1107/S2056989024000756/jp2002Isup3.cdx

Supporting information file. DOI: 10.1107/S2056989024000756/jp2002Isup4.cml

CCDC reference: 2327435

Additional supporting information:  crystallographic information; 3D view; checkCIF report

## Figures and Tables

**Table 1:** Hydrogen-bond geometry (Å, °) *Cg*3 is the centroid of the C10–C15 benzene ring.

*D*—H⋯*A*	*D*—H	H⋯*A*	*D*⋯*A*	*D*—H⋯*A*
C4—H4⋯O2^i^	0.95	2.41	3.2192 (16)	142
C9—H9*A*⋯*Cg*3^ii^	0.99	2.61	3.4936 (15)	148
C9—H9*B*⋯O2^iii^	0.99	2.58	3.5208 (17)	158
C16—H16*B*⋯*Cg*3^iv^	0.98	2.85	3.5685 (16)	131

Symmetry codes: (i) 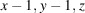 ; (ii) 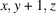 ; (iii) 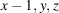 ; (iv) 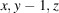 .

**Table 2:** Comparison of the selected (X-ray and DFT) geometric data (Å, °)

Bonds/angles	X-ray	B3LYP/6-311G(d,p)
O1—C7	1.2094 (16)	1.253
O2—C8	1.2110 (15)	1.242
N1—C7	1.3684 (16)	1.381
N1—C6	1.4108 (15)	1.395
N1—C9	1.4610 (15)	1.510
C1—C2	1.3835 (17)	1.414
C7—N1—C6	110.91 (10)	110.6
C7—N1—C9	124.13 (11)	124.25
C6—N1—C9	124.65 (10)	124.85
O1—C7—N1	127.83 (12)	127.65
O1—C7—C8	126.45 (12)	129.51
N1—C7—C8	105.72 (10)	105.60
O2—C8—C1	130.74 (13)	130.12
C7—N1—C9—C10	114.84 (13)	113.99
N1—C9—C10—C11	125.40 (13)	125.86
C7—N1—C9—C10	114.84 (13)	114.23
N1—C7—C8—O2	178.78 (12)	178.52
O1—C7—C8—C1	178.80 (13)	178.36

**Table 3:** Experimental details

Crystal data	
Chemical formula	C_16_H_13_NO_2_
*M*_r_	251.27
Crystal system, space group	Monoclinic, *P*2_1_/*c*
Temperature (K)	150
*a*, *b*, *c* (Å)	6.6126 (4), 4.8680 (3), 38.924 (2)
β (°)	94.118 (2)
*V* (Å^3^)	1249.74 (13)
*Z*	4
Radiation type	Mo *K*α
μ (mm^−1^)	0.09
Crystal size (mm)	0.37 × 0.29 × 0.03

Data collection	
Diffractometer	Bruker D8 QUEST PHOTON 3
Absorption correction	Numerical (*SADABS*; Krause *et al.*, 2015 ▸)
*T*_min_, *T*_max_	0.97, 1.00
No. of measured, independent and observed [*I* > 2σ(*I*)] reflections	41604, 4846, 3359
*R*_int_	0.056
(sin θ/λ)_max_ (Å^−1^)	0.773

Refinement	
*R*[*F* ^2^ > 2σ(*F* ^2^)], *wR*(*F* ^2^), *S*	0.054, 0.137, 1.03
No. of reflections	4846
No. of parameters	173
H-atom treatment	H-atom parameters constrained
Δρ_max_, Δρ_min_ (e Å^−3^)	0.30, −0.22

Computer programs: *APEX4* (Bruker, 2021 ▸), *SAINT* (Bruker, 2021 ▸), *SHELXT* (Sheldrick, 2015*a* ▸), *SHELXL2018* (Sheldrick, 2015*b* ▸), *DIAMOND* (Brandenburg & Putz, 2012 ▸) and *SHELXTL* (Bruker, 2021 ▸).

## supplementary crystallographic information

### Crystal data

**Table:**

C_16_H_13_NO_2_	*F*(000) = 528
*M**_r_* = 251.27	*D*_x_ = 1.335 Mg m^−^^3^
Monoclinic, *P*2_1_/*c*	Mo *K*α radiation, λ = 0.71073 Å
*a* = 6.6126 (4) Å	Cell parameters from 9960 reflections
*b* = 4.8680 (3) Å	θ = 3.1–32.6°
*c* = 38.924 (2) Å	µ = 0.09 mm^−^^1^
β = 94.118 (2)°	*T* = 150 K
*V* = 1249.74 (13) Å^3^	Plate, orange
*Z* = 4	0.37 × 0.29 × 0.03 mm

### Data collection

**Table:**

Bruker D8 QUEST PHOTON 3 diffractometer	4846 independent reflections
Radiation source: fine-focus sealed tube	3359 reflections with *I* > 2σ(*I*)
Graphite monochromator	*R*_int_ = 0.056
Detector resolution: 7.3910 pixels mm^-1^	θ_max_ = 33.3°, θ_min_ = 2.1°
φ and ω scans	*h* = −10→10
Absorption correction: numerical (*SADABS*; Krause *et al.*, 2015)	*k* = −7→7
*T*_min_ = 0.97, *T*_max_ = 1.00	*l* = −59→60
41604 measured reflections	

### Refinement

**Table:**

Refinement on *F*^2^	Primary atom site location: dual
Least-squares matrix: full	Secondary atom site location: difference Fourier map
*R*[*F*^2^ > 2σ(*F*^2^)] = 0.054	Hydrogen site location: inferred from neighbouring sites
*wR*(*F*^2^) = 0.137	H-atom parameters constrained
*S* = 1.03	*w* = 1/[σ^2^(*F*_o_^2^) + (0.050*P*)^2^ + 0.4692*P*] where *P* = (*F*_o_^2^ + 2*F*_c_^2^)/3
4846 reflections	(Δ/σ)_max_ = 0.001
173 parameters	Δρ_max_ = 0.30 e Å^−^^3^
0 restraints	Δρ_min_ = −0.22 e Å^−^^3^

### Special details

**Table:**

Experimental. The diffraction data were obtained from 7 sets of frames, each of width 0.5° in ω, collected with scan parameters determined by the "strategy" routine in *APEX4*. The scan time was 7.5 sec/frame.
Geometry. All esds (except the esd in the dihedral angle between two l.s. planes) are estimated using the full covariance matrix. The cell esds are taken into account individually in the estimation of esds in distances, angles and torsion angles; correlations between esds in cell parameters are only used when they are defined by crystal symmetry. An approximate (isotropic) treatment of cell esds is used for estimating esds involving l.s. planes.
Refinement. Refinement of F^2^ against ALL reflections. The weighted R-factor wR and goodness of fit S are based on F^2^, conventional R-factors R are based on F, with F set to zero for negative F^2^. The threshold expression of F^2^ > 2sigma(F^2^) is used only for calculating R-factors(gt) etc. and is not relevant to the choice of reflections for refinement. R-factors based on F^2^ are statistically about twice as large as those based on F, and R- factors based on ALL data will be even larger. H-atoms attached to carbon were placed in calculated positions (C—H = 0.95 - 0.99 Å). All were included as riding contributions with isotropic displacement parameters 1.2 - 1.5 times those of the attached atoms.

### Fractional atomic coordinates and isotropic or equivalent isotropic displacement parameters (Å^2^)

**Table:**

	*x*	*y*	*z*	*U*_iso_*/*U*_eq_	
O1	0.83652 (16)	1.2777 (2)	0.62976 (3)	0.0407 (3)	
O2	1.08789 (14)	1.0379 (2)	0.68782 (3)	0.0388 (2)	
N1	0.60620 (15)	0.9544 (2)	0.64653 (3)	0.0256 (2)	
C1	0.78827 (17)	0.7578 (2)	0.69329 (3)	0.0252 (2)	
C2	0.82166 (19)	0.5820 (3)	0.72105 (3)	0.0288 (2)	
H2	0.946524	0.584733	0.734737	0.035*	
C3	0.6679 (2)	0.4016 (3)	0.72839 (3)	0.0303 (3)	
H3	0.686776	0.278853	0.747327	0.036*	
C4	0.48593 (19)	0.4006 (3)	0.70795 (3)	0.0286 (2)	
H4	0.382752	0.274792	0.713208	0.034*	
C5	0.45014 (18)	0.5784 (3)	0.67999 (3)	0.0258 (2)	
H5	0.324978	0.576843	0.666387	0.031*	
C6	0.60429 (17)	0.7564 (2)	0.67298 (3)	0.0234 (2)	
C7	0.78645 (19)	1.0935 (3)	0.64820 (3)	0.0291 (2)	
C8	0.91639 (18)	0.9665 (3)	0.67911 (3)	0.0288 (3)	
C9	0.43199 (19)	1.0233 (3)	0.62289 (3)	0.0286 (2)	
H9A	0.461089	1.195878	0.610731	0.034*	
H9B	0.312938	1.056262	0.636366	0.034*	
C10	0.37999 (19)	0.8018 (3)	0.59657 (3)	0.0266 (2)	
C11	0.1851 (2)	0.6927 (3)	0.59296 (3)	0.0335 (3)	
H11	0.086091	0.753530	0.607771	0.040*	
C12	0.1334 (2)	0.4962 (3)	0.56801 (4)	0.0364 (3)	
H12	−0.000569	0.424591	0.565979	0.044*	
C13	0.2747 (2)	0.4027 (3)	0.54597 (3)	0.0337 (3)	
C14	0.4703 (2)	0.5080 (3)	0.55005 (3)	0.0339 (3)	
H14	0.570145	0.443987	0.535606	0.041*	
C15	0.5222 (2)	0.7055 (3)	0.57492 (3)	0.0305 (3)	
H15	0.656602	0.775497	0.577128	0.037*	
C16	0.2171 (3)	0.1907 (3)	0.51890 (4)	0.0460 (4)	
H16A	0.120928	0.270980	0.501370	0.069*	
H16B	0.154047	0.033158	0.529600	0.069*	
H16C	0.338779	0.129939	0.508103	0.069*	

### Atomic displacement parameters (Å^2^)

**Table:**

	*U*^11^	*U*^22^	*U*^33^	*U*^12^	*U*^13^	*U*^23^
O1	0.0409 (6)	0.0403 (6)	0.0417 (6)	−0.0093 (4)	0.0079 (4)	0.0080 (4)
O2	0.0248 (4)	0.0454 (6)	0.0457 (6)	−0.0087 (4)	−0.0005 (4)	−0.0040 (5)
N1	0.0258 (5)	0.0251 (5)	0.0253 (5)	−0.0025 (4)	−0.0012 (4)	0.0006 (4)
C1	0.0232 (5)	0.0254 (5)	0.0268 (5)	−0.0004 (4)	−0.0002 (4)	−0.0036 (4)
C2	0.0279 (6)	0.0286 (6)	0.0289 (6)	0.0013 (5)	−0.0043 (4)	−0.0024 (5)
C3	0.0348 (6)	0.0269 (6)	0.0287 (6)	0.0013 (5)	−0.0013 (5)	0.0016 (5)
C4	0.0308 (6)	0.0259 (5)	0.0293 (6)	−0.0042 (5)	0.0035 (5)	−0.0010 (5)
C5	0.0246 (5)	0.0256 (5)	0.0270 (5)	−0.0019 (4)	−0.0003 (4)	−0.0027 (4)
C6	0.0234 (5)	0.0229 (5)	0.0236 (5)	0.0001 (4)	0.0001 (4)	−0.0032 (4)
C7	0.0284 (6)	0.0295 (6)	0.0299 (6)	−0.0040 (5)	0.0047 (5)	−0.0017 (5)
C8	0.0245 (5)	0.0305 (6)	0.0313 (6)	−0.0024 (5)	0.0021 (4)	−0.0056 (5)
C9	0.0297 (6)	0.0279 (6)	0.0275 (6)	0.0013 (5)	−0.0032 (4)	0.0008 (4)
C10	0.0294 (6)	0.0264 (5)	0.0233 (5)	−0.0013 (4)	−0.0030 (4)	0.0028 (4)
C11	0.0289 (6)	0.0394 (7)	0.0320 (6)	−0.0024 (5)	−0.0006 (5)	0.0022 (5)
C12	0.0327 (6)	0.0393 (7)	0.0358 (7)	−0.0103 (6)	−0.0073 (5)	0.0036 (6)
C13	0.0448 (7)	0.0274 (6)	0.0273 (6)	−0.0057 (5)	−0.0080 (5)	0.0036 (5)
C14	0.0401 (7)	0.0338 (6)	0.0276 (6)	−0.0017 (5)	0.0006 (5)	−0.0014 (5)
C15	0.0298 (6)	0.0328 (6)	0.0286 (6)	−0.0048 (5)	0.0002 (5)	0.0005 (5)
C16	0.0660 (10)	0.0355 (7)	0.0342 (7)	−0.0107 (7)	−0.0118 (7)	−0.0005 (6)

### Geometric parameters (Å, º)

**Table:**

O1—C7	1.2094 (16)	C9—C10	1.5098 (17)
O2—C8	1.2110 (15)	C9—H9A	0.9900
N1—C7	1.3684 (16)	C9—H9B	0.9900
N1—C6	1.4108 (15)	C10—C15	1.3886 (18)
N1—C9	1.4610 (15)	C10—C11	1.3917 (18)
C1—C2	1.3835 (17)	C11—C12	1.388 (2)
C1—C6	1.4026 (16)	C11—H11	0.9500
C1—C8	1.4567 (18)	C12—C13	1.390 (2)
C2—C3	1.3886 (18)	C12—H12	0.9500
C2—H2	0.9500	C13—C14	1.390 (2)
C3—C4	1.3943 (17)	C13—C16	1.5040 (19)
C3—H3	0.9500	C14—C15	1.3895 (18)
C4—C5	1.3973 (17)	C14—H14	0.9500
C4—H4	0.9500	C15—H15	0.9500
C5—C6	1.3798 (17)	C16—H16A	0.9800
C5—H5	0.9500	C16—H16B	0.9800
C7—C8	1.5555 (18)	C16—H16C	0.9800
			
C7—N1—C6	110.91 (10)	C10—C9—H9A	108.9
C7—N1—C9	124.13 (11)	N1—C9—H9B	108.9
C6—N1—C9	124.65 (10)	C10—C9—H9B	108.9
C2—C1—C6	121.38 (11)	H9A—C9—H9B	107.7
C2—C1—C8	131.50 (11)	C15—C10—C11	118.21 (12)
C6—C1—C8	107.12 (11)	C15—C10—C9	121.45 (11)
C1—C2—C3	118.28 (11)	C11—C10—C9	120.33 (12)
C1—C2—H2	120.9	C12—C11—C10	120.87 (13)
C3—C2—H2	120.9	C12—C11—H11	119.6
C2—C3—C4	119.96 (12)	C10—C11—H11	119.6
C2—C3—H3	120.0	C11—C12—C13	121.00 (13)
C4—C3—H3	120.0	C11—C12—H12	119.5
C3—C4—C5	122.23 (12)	C13—C12—H12	119.5
C3—C4—H4	118.9	C12—C13—C14	118.03 (12)
C5—C4—H4	118.9	C12—C13—C16	120.64 (13)
C6—C5—C4	117.18 (11)	C14—C13—C16	121.32 (14)
C6—C5—H5	121.4	C15—C14—C13	121.06 (13)
C4—C5—H5	121.4	C15—C14—H14	119.5
C5—C6—C1	120.98 (11)	C13—C14—H14	119.5
C5—C6—N1	128.22 (11)	C10—C15—C14	120.81 (12)
C1—C6—N1	110.80 (10)	C10—C15—H15	119.6
O1—C7—N1	127.83 (12)	C14—C15—H15	119.6
O1—C7—C8	126.45 (12)	C13—C16—H16A	109.5
N1—C7—C8	105.72 (10)	C13—C16—H16B	109.5
O2—C8—C1	130.74 (13)	H16A—C16—H16B	109.5
O2—C8—C7	123.82 (12)	C13—C16—H16C	109.5
C1—C8—C7	105.44 (10)	H16A—C16—H16C	109.5
N1—C9—C10	113.23 (10)	H16B—C16—H16C	109.5
N1—C9—H9A	108.9		
			
C6—C1—C2—C3	0.24 (18)	C2—C1—C8—C7	−179.07 (13)
C8—C1—C2—C3	−179.89 (12)	C6—C1—C8—C7	0.82 (13)
C1—C2—C3—C4	0.10 (19)	O1—C7—C8—O2	−1.4 (2)
C2—C3—C4—C5	−0.5 (2)	N1—C7—C8—O2	178.78 (12)
C3—C4—C5—C6	0.58 (18)	O1—C7—C8—C1	178.80 (13)
C4—C5—C6—C1	−0.23 (17)	N1—C7—C8—C1	−1.02 (13)
C4—C5—C6—N1	−179.90 (11)	C7—N1—C9—C10	114.84 (13)
C2—C1—C6—C5	−0.17 (18)	C6—N1—C9—C10	−72.24 (15)
C8—C1—C6—C5	179.93 (11)	N1—C9—C10—C15	−55.98 (16)
C2—C1—C6—N1	179.55 (11)	N1—C9—C10—C11	125.40 (13)
C8—C1—C6—N1	−0.35 (13)	C15—C10—C11—C12	−1.03 (19)
C7—N1—C6—C5	179.35 (12)	C9—C10—C11—C12	177.63 (12)
C9—N1—C6—C5	5.62 (19)	C10—C11—C12—C13	0.1 (2)
C7—N1—C6—C1	−0.34 (14)	C11—C12—C13—C14	1.1 (2)
C9—N1—C6—C1	−174.07 (11)	C11—C12—C13—C16	−179.70 (13)
C6—N1—C7—O1	−178.98 (13)	C12—C13—C14—C15	−1.4 (2)
C9—N1—C7—O1	−5.2 (2)	C16—C13—C14—C15	179.46 (13)
C6—N1—C7—C8	0.84 (13)	C11—C10—C15—C14	0.78 (19)
C9—N1—C7—C8	174.60 (11)	C9—C10—C15—C14	−177.87 (12)
C2—C1—C8—O2	1.2 (2)	C13—C14—C15—C10	0.4 (2)
C6—C1—C8—O2	−178.96 (14)		

### Hydrogen-bond geometry (Å, º)

*Cg*3 is the centroid of the C10···C15 benzene ring.

**Table:**

*D*—H···*A*	*D*—H	H···*A*	*D*···*A*	*D*—H···*A*
C4—H4···O2^i^	0.95	2.41	3.2192 (16)	142
C9—H9*A*···*Cg*3^ii^	0.99	2.61	3.4936 (15)	148
C9—H9*B*···O2^iii^	0.99	2.58	3.5208 (17)	158
C16—H16*B*···*Cg*3^iv^	0.98	2.85	3.5685 (16)	131

Symmetry codes: (i) *x*−1, *y*−1, *z*; (ii) *x*, *y*+1, *z*; (iii) *x*−1, *y*, *z*; (iv) *x*, *y*−1, *z*.
